# Automatic Recognition Algorithm for Renal Tumors and Cysts in CT Images using Mamba and YOLO11

**DOI:** 10.2174/0115734056450311260425112727

**Published:** 2026-04-28

**Authors:** Jiali Jiang, Zhaoxue Chen

**Affiliations:** 1 School of Health Science and Engineering, University of Shanghai for Science and Technology, Shanghai 200000, China

**Keywords:** Renal tumor, YOLO11 model, Mamba, Deep learning, CT image, Medical intelligence

## Abstract

**Introduction:**

Renal tumors pose a serious threat to patient health and survival, highlighting the importance of early detection and accurate diagnosis. In clinical practice, differentiating renal tumors from cysts in CT images remains challenging due to similar imaging characteristics and complex anatomical structures. The aim of this study is to develop an improved detection method for renal tumors and cysts based on an enhanced YOLO11 framework.

**Methods:**

An improved YOLO11-based detection model incorporating a Mamba-inspired architecture is proposed. A gated state-space modeling module is introduced into the backbone network to enhance the modeling capability of spatial and channel information and effectively focus on key regional features. A Dynamic Upsampling module (DySample) is then adopted in the neck network to improve multi-scale feature fusion. In addition, a Multi-Dimensional Local Channel Attention (MLCA) mechanism is integrated before the detection head to jointly refine spatial and channel features, thereby enhancing the localization capability for lesion areas.

**Results:**

Experimental results demonstrate that the proposed method achieves a precision of 0.837, a recall of 0.636, mAP@0.5 of 0.732, and mAP@0.5:0.95 of 0.505. Compared with the YOLO11 model, these metrics are improved by 3.1%, 0.1%, 2.1%, and 2.5%, respectively, indicating an overall enhancement in detection performance.

**Discussion:**

YOLO11-Mamba has achieved improvements in detection accuracy and localization performance, but there are still some potential limitations. Among these, the introduction of state space models and attention mechanisms has increased the model's parameter count and computational complexity to some extent, which may pose challenges for clinical deployment, pointing the way for future research.

**Conclusion:**

The proposed method demonstrates effective performance in the detection of renal tumors and cysts from CT images. The results show fewer missed detections and improved lesion localization accuracy, suggesting the proposed model is a promising tool for renal lesion detection and clinical imaging.

## 1 INTRODUCTION

Renal tumors are a common type of urinary system growth, with cases increasing over the past few decades and posing a serious risk to patient health. Detecting and diagnosing these tumors early is crucial for successful treatment and a better prognosis for patients, guiding surgery, preserving kidney function, and preventing tumor spread [1]. Renal cysts are fluid-filled sacs that form on the surface or inside the kidney. Single renal cysts are common and can be associated with issues in the kidney or blood function. As a major abdominal organ, the kidney has complex borders that distinguish it from tumors and cysts. Overlapping tissues make it difficult to distinguish between the two [2]. Currently, CT image provides clear pictures of conditions related to renal tumors, help locate and accurately describe tumors, and are a key tool for doctors in diagnosing early-stage disease.

With the rapid development of deep learning, an increasing number of studies have focused on applying neural networks to computer vision tasks in medical imaging. Zhao *et al.* [3] proposed a Convolutional Neural Network (CNN)-based U-Net architecture that incorporates attention mechanisms for renal tumor detection, enabling automatic kidney segmentation without manual intervention. Their experimental results demonstrated the capability of the proposed framework to distinguish tumor-bearing kidneys and support early tumor detection. Praveen *et al.* [4] employed a deep CNN for renal tumor identification by integrating Residual Network (ResNet) and ResNeXt architectures, achieving improved detection accuracy. Alzu’bi *et al.* [5] trained three deep learning models using CT images to distinguish renal tumors, achieving classification accuracies of up to 97%, although the proposed framework involved relatively complex model structures. Bingol *et al.* [6] developed a novel hybrid model for classifying CT images containing renal stones, tumors, and cysts, reporting a high accuracy of 99.37%. Zabihollahy *et al.* [7] investigated the use of deep learning for detecting solid renal masses on CT and achieved an accuracy of 83.75% in distinguishing malignant from benign tumors. These studies demonstrate the feasibility and effectiveness of deep learning methods for renal tumor detection and classification in CT imaging.

In medical image analysis, deep learning-based renal tumor detection methods generally fall into two categories: semantic segmentation and object detection. The YOLO (You Only Look Once) series represents a typical object detection framework that performs object localization and classification through a single forward pass, simultaneously predicting bounding boxes and class probabilities. Due to its high computational efficiency, YOLO has attracted considerable attention in medical imaging applications, including the detection and localization of anatomical structures, lesions, and tumors. Previous studies have reported the favorable performance of YOLO-based methods in renal tumor detection using Magnetic Resonance Imaging (MRI). For example, Fouziya *et al.* [8] evaluated the performance of YOLOv9 on large-scale renal tumor datasets and demonstrated its ability to effectively distinguish tumors from renal structures in MRI images. Anari *et al.* [9] applied YOLOv7 for renal cancer detection in excretory-phase MRI images, achieving a positive predictive value and sensitivity of 0.75 and 0.69, respectively. Pande and Agarwal [10] employed a YOLOv8n-cls model for the rapid and high-confidence detection of multiple renal diseases and proposed a customizable clinical diagnostic platform. However, existing studies have primarily focused on MRI data, and the application of YOLO-based methods to CT images, particularly for renal tumor and cyst analysis, remains relatively limited. Although YOLO models exhibit high computational efficiency in feature extraction through traditional convolutional backbone networks, their receptive fields are inherently limited, and they lack the ability to effectively model long-range dependencies. As a result, capturing distant contextual information in complex abdominal CT images remains challenging. This limitation becomes particularly pronounced in abdominal CT imaging, where anatomical structures are irregular and diverse, demanding superior non-local and multi-scale modeling capabilities from detection algorithms.

To address these limitations, this study introduces an enhanced mechanism based on the selective state space model Mamba [11], which features linear computational complexity and strong long-range dependency modeling capability. The proposed method aims to enhance the detection performance of YOLO in complex renal CT images while maintaining high inference efficiency. By incorporating the Mamba mechanism, the proposed framework enables more accurate identification and localization of renal tumors and cysts. The main contributions of this study can be summarized as follows: (1) A gated structure-aware state-space module is introduced into the backbone network to enhance spatial and channel feature representation and achieve more effective focus on critical regions. (2) A Dynamic Upsampling (DySample) module is employed in the neck network to improve multi-scale feature fusion and optimize detection performance across lesion sizes. (3) A Multi-Dimensional Local Channel Attention (MLCA) mechanism is embedded prior to the detection head to jointly refine spatial and channel information, resulting in enhanced localization accuracy for renal tumors and cysts.

## 2 RELATED WORK

YOLO11 [12], released by Ultralytics in 2024, is an improved object detection model designed to achieve higher detection accuracy with a more lightweight network structure compared with YOLOv8. The overall architecture of YOLO11 consists of four main components: input layer, backbone network, neck network, and output layer. In the input stage, data augmentation and preprocessing operations, such as adaptive anchor assignment and image resizing, are applied to enhance model robustness. The backbone network is responsible for extracting semantic features from the input images. In YOLO11, the C2f module is replaced by the C3K2 module, improving feature extraction efficiency and reducing computational cost. In addition, a C2PSA module is introduced after the Spatial Pyramid Pooling Fast (SPPF) layer to further enhance multi-scale feature representation. In the neck network, a bidirectional feature pyramid structure is employed to enable cross-scale feature fusion, allowing deep semantic information to be propagated to shallow layers while simultaneously aggregating fine-grained spatial information into deeper features. The output layer adopts a decoupled detection head with depthwise separable convolutions incorporated into the classification branch, optimizing the simultaneous execution of object detection and classification tasks.

The Mamba model was initially developed for processing one-dimensional sequential data, such as text and audio, and has demonstrated strong performance in natural language processing tasks. More recently, Mamba has been successfully extended to the vision domain. Liu *et al.* [13] proposed the Vision Mamba (VMamba) model in 2024, which leverages the advantages of State Space Models (SSM) and introduces a two-dimensional selective scanning mechanism (2D Selective-Scan, SS2D) to integrate image information from multiple spatial directions. This approach has shown competitive performance in image classification tasks. Subsequently, Chen *et al.* [14] further optimized VMamba and proposed the Res-VMamba model, achieving state-of-the-art results in fine-grained food classification. Wang *et al.* [15] introduced the Mamba-YOLO model, which incorporates a novel SSM design for real-time object detection. In addition, Cao *et al.* [16] integrated an optimized SSM into the YOLO framework, effectively exploiting both local and global dependency modeling capabilities of SSM to improve detection performance.

## 3 METHODS AND MATERIALS

### 3.1 Model Architecture

In this study, YOLO11 is adopted as the baseline model, and the backbone network is redesigned by incorporating an EVSSBlock module to enhance spatial information representation. To further improve feature expression for renal tumors and cyst regions, DySample is introduced into the neck network, enabling more effective multi-scale feature fusion. In addition, the MLCA mechanism is integrated before the detection head to integrate channel and spatial information, thereby improving detection accuracy for small, medium, and large lesions under complex background conditions. The overall architecture of the improved model is illustrated in Fig. (**1**).

### 3.2 Backbone

The YOLO11 architecture relies on conventional convolutional operations. Although this design provides strong local feature extraction and high inference efficiency, it remains limited in handling long-range dependencies and in effectively representing multi-scale targets. To address these limitations, this study introduces the EVSSBlock module to redesign and enhance the backbone network. The improved backbone consists of five main components: the Stem module, VisionClueMerge, EVSSBlock, SPPF, and C2PSA. The Stem module uses a series of convolution, downsampling, batch normalization, and activation operations to extract multi-level features from the input images, as illustrated in Fig. (**2**).

VisionClueMerge [16] divides the input feature maps into non-overlapping patches and strengthens feature representation by concatenating information across diverse spatial locations. This approach increases feature dimensionality through the combination of multiple spatial positions while maintaining spatial details, thereby improving the model’s capacity to perceive spatial information. Inspired by the design principles of VMamba, the proposed EVSSBlock is constructed based on the VSSBlock architecture while retaining the SS2D. EVSSBlock is built on a structure-aware SSM and adopts a linear-complexity visual representation learning strategy to effectively capture cross-layer dependencies. This design addresses the limitations of conventional convolutional networks in modeling global structural relationships among targets in complex medical images. The EVSSBlock module comprises three primary components: ECBlock, RGBlock, and SS2D, as shown in Fig. (**3**).

The ECBlock captures information at multiple scales by employing parallel depthwise separable convolutions with kernel sizes of 1, 3, and 5. Subsequently, an ECA mechanism is applied to enhance informative channel features. The resulting features are then combined with the residual input through element-wise addition to produce the final output, as described in (Eqs. **1**-**4**). This design enables effective extraction of local spatial information while maintaining computational efficiency and enhancing feature representation capability. The features are fused with the original input *via* residual connections, allowing the model to preserve original information while further capturing complex pattern characteristics.


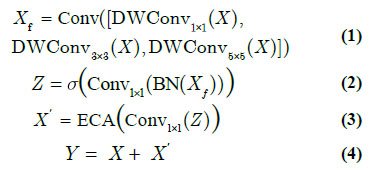


In these equations, *X* denotes the input feature map, *X_f_* represents the concatenated multi-scale features along the channel dimension, *BN* denotes batch normalization, σ represents the activation function, and *Y* denotes the output feature map.

The RGBlock constructs two parallel branches at the input stage. One branch performs channel expansion through a convolution operation with a kernel size of 1, enabling feature recalibration, while the other branch employs depthwise separable convolution to form a positional encoding module that effectively captures local spatial features. Feature decoupling is achieved through a channel-splitting strategy, and a gating mechanism dynamically fuses features from the two branches, as described in (Eqs. **5**-**8**). The RGBlock adopts the Gaussian Error Linear Unit (GELU) activation function and integrates features through element-wise multiplication with the complementary branch. Channel information is further refined using convolution and global feature aggregation, followed by feature summation to generate the final output. *X_1_* and *X_2_* denote the input feature map, and *Y* denotes the output feature map.


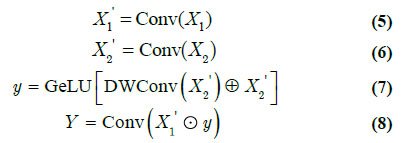


### 3.3 Dynamic Upsampling

Renal CT images often contain numerous small tumors or cystic lesions. Traditional upsampling methods typically rely on bilinear interpolation, which operates through fixed mechanisms. Upsampling methods function similarly to mechanically magnifying a grid-based image. Based on existing pixel values in the original grid, they use predetermined rules, such as averaging neighboring pixels, to infer pixel values for the newly created blank areas in the enlarged image. However, this approach can result in the loss of substantial high-frequency features such as edges and textures, as it struggles to preserve fine image details and boundary information. To address these limitations, this study adopts a DySample [17] that performs adaptive sampling based on points while avoiding computationally expensive dynamic convolution operations. The core idea of DySample is to expand each input feature point into multiple upsampling points and to adaptively search for optimal semantic aggregation locations for each point, thereby enabling efficient feature mapping and fusion. The overall structure of the DySample module is illustrated in Fig. (**4**).

Specifically, the input feature map *X* is represented as a tensor of size (C, H, W). Given an upsampling scale factor s, a linear transformation with 2s^2^ output channels to generate an offset map O, which determines the spatial positions of newly generated points during the upsampling process, as shown in (Eq. **9**). The offset map has a spatial size of 2s^2^×H×W. It is subsequently reshaped through a pixel rearrangement operation into a tensor of size 2s×sH×sW. The resulting point sampling set S is defined as the sum of the offset map O and the initial sampling grid G, where g denotes the number of channel groups, as described in (Eq. **10**). Finally, the input feature map *X* is resampled according to the coordinates defined in the set S, producing an upsampled feature map *X'* with enhanced resolution and a spatial size of (C,sH,sW) in (Eq. **11**).


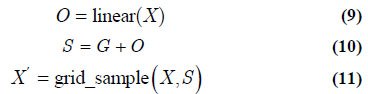


### 3.4 Mixed Local Channel Attention

To enhance the representation of multi-scale features in lesion regions, this study introduces a lightweight MLCA mechanism [18]. By jointly using channel-wise and spatial information, the MLCA module integrates local features with global contextual cues to enhance feature representation. Specifically, the input feature map is initially processed using Local Average Pooling (LAP) and is subsequently split into two parallel branches. In the first branch, Global Average Pooling (GAP) is applied to the LAP output to extract global contextual features. In the second branch, the LAP output is reshaped into a tensor of dimensions (1,1,C)×ks×ks to retain local spatial information. Following this, both branches use one-dimensional convolution for feature transformation, followed by unpooling operations to restore the original resolution, after which the transformed features are fused. Finally, the fused features are mapped to the original spatial dimensions through an additional unpooling operation and are combined with their original feature map *via* element-wise multiplication. This approach enables the joint modeling of local and global attention, thereby enhancing the model’s capacity to capture lesion characteristics across various scales. The overall architecture of the MLCA module is illustrated in Fig. (**5**).

### 3.5 Dataset

The public KiTS23 dataset was used for model training and internal validation. This dataset comprises 489 CT scans with expert annotations of renal tumors and cysts [19]. The KiTS23 dataset includes scans acquired during the corticomedullary and nephrogenic phases, with slice thicknesses ranging from 0.5 mm to 5.0 mm. To construct the experimental dataset, a two-dimensional (2D) slice selection strategy was adopted, centered on the maximal axial cross-section of each lesion. Specifically, for each tumor or cyst, the axial slice with the largest lesion cross-sectional area was first identified based on the three-dimensional annotations provided in the KiTS23 dataset and selected as the central slice. On this basis, neighboring axial slices at offsets of ±2 and ±4 relative to the central slice were further extracted to cover local spatial variations of the lesion along the axial direction. When a patient presented with multiple tumors or cysts and lesion regions overlapped within the same image, the corresponding slices were merged. As a result, each case yielded 5–10 two-dimensional slices containing valid lesion information. Using this strategy, all lesions were projected and annotated on their corresponding 2D slices according to the ground-truth labels. After data augmentation, the final dataset comprised 3,009 images. These images were randomly divided into training, validation, and testing subsets. The training set included 2,418 images, the validation set contained 298 images, and the testing set consisted of 293 images.

### 3.6 Experimental Setup

All experiments were implemented using the PyTorch deep learning framework. Model training was conducted on a workstation equipped with an AMD EPYC 9654 CPU and an NVIDIA GeForce RTX 4090 GPU, with Python 3.8 used as the programming language. During training, input images were resized to a resolution of 640×640 pixels, and the network was trained for 300 epochs. A batch size of 12 was adopted, and a dynamic learning rate strategy was applied, with an initial learning rate set to 0.01.

### 3.7 Evaluation Metrics

Model performance was evaluated in terms of both detection accuracy and model complexity. Detection accuracy was assessed using Precision, Recall, and mean Average Precision (mAP). The prediction results were classified into True Positive (TP), False Negative (FN), False Positive (FP), and True Negative (TN). The formulas for Precision and Recall are presented in Eqs. (**12**) and (**13**). Average Precision (AP) is a comprehensive metric that reflects detection performance across various confidence thresholds, as defined in Eq. (**14**). The mAP was calculated as the average AP across all categories based on the Precision–Recall curves Eq. (**15**). Specifically, mAP@0.5 refers to the mAP at an Intersection over Union (IoU) threshold of 0.5, whereas mAP@0.5:0.95 represents the average mAP across IoU thresholds ranging from 0.5 to 0.95 with a step size of 0.05. Model complexity was evaluated based on computational cost (GFLOPs) and model size.

Comparative experiments between YOLO11-Mamba and the baseline YOLO11s were repeated three times under identical training settings with different random seeds. The results are presented as mean±standard deviation, and paired t-tests were conducted to assess statistical significance, with *p* < 0.05 considered statistically significant.


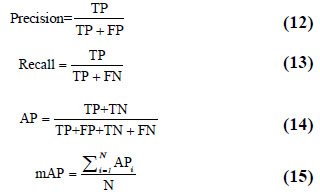


## 4 RESULTS AND DISCUSSION

### 4.1. Performance Comparison with Other Models

To evaluate the performance of the proposed model in renal tumor and cyst detection, YOLO11s was selected as the baseline for systematic experiments. After training and validation on the dataset, the proposed method was compared with several object detection approaches, and the results are summarized in Table **1**.

As shown in Table **1**, YOLO11-Mamba achieves the highest Precision of 0.837 and mAP@0.5 of 0.732 among all models, outperforming all baseline detectors and Mamba-YOLO. It also delivers a competitive Recall of 0.636 and the highest mAP@0.5:0.95 of 0.505, demonstrating its robust performance across all key accuracy metrics. Compared with Mamba-YOLO, YOLO11-Mamba achieves an absolute increase of 0.025 in precision, 0.007 in mAP@0.5, and 0.010 in mAP@0.5:0.95, while reducing GFLOPs by 16.8G. Compared to YOLO11s, YOLO11-Mamba improves Precision by 0.031, mAP@0.5 by 0.021, and mAP@0.5:0.95 by 0.025, with a slight increase in parameters and a reduction in inference speed. Notably, YOLO11-Mamba is the only model among these three to achieve an mAP@0.5:0.95 exceeding 0.50, indicating its robust detection capability across diverse IoU thresholds.

Among the baseline YOLO series, YOLO11-Mamba achieves 0.013 and 0.016 higher mAP@0.5 than YOLOv5s and YOLOv8s, respectively, along with 0.024 higher mAP@0.5:0.95 over both models. The proposed model also outperforms YOLOv10s by 0.018 in mAP@0.5 and 0.027 in mAP@0.5:0.95, yielding superior accuracy despite YOLOv10’s lightweight design. Furthermore, it surpasses YOLOv6s by 0.037 in mAP@0.5 and 0.035 in mAP@0.5:0.95. YOLO11-Mamba improves precision by 0.032 over YOLOv10s and has 2.90M fewer parameters than YOLOv6s, demonstrating a favorable balance between accuracy and efficiency. Compared with YOLO variants, integrating the Mamba mechanism enhances performance, validating its effectiveness in capturing long-range dependencies and non-local feature interactions.

Compared with two-stage detectors, the proposed YOLO11-Mamba model shows significant gains: it improves mAP@0.5 by 0.222 over Cascade R-CNN and by 0.183 over Faster R-CNN, while simultaneously reducing parameters by 39.16M and 11.35M, and GFLOPs by 129.1G and 101.1G, respectively. By contrast, two-stage detectors provide reasonable detection capability but require larger model sizes and higher computational cost, which may limit their applicability in clinical settings. Their overall detection performance is also lower than that of the one-stage detection framework adopted in this study.

Furthermore, YOLO11-Mamba demonstrates superior performance relative to both SwinTransformer-YOLO and RT-DETR. It achieves significant improvements in detection accuracy, with gains of 0.036 and 0.068 in mAP@0.5, and 0.035 and 0.063 in mAP@0.5:0.95, respectively. Moreover, its precision exceeds that of the two counterparts by 0.046 and 0.066. These accuracy improvements are attained while simultaneously enhancing model efficiency. YOLO11-Mamba reduces parameter counts by 37.80M and 36.10M, and more substantially, lowers computational costs by 374.5G and 75.1G GFLOPs. Its computational complexity is also drastically reduced. The proposed model employs SSM to achieve global context modeling with linear computational complexity, enabling favorable detection performance while maintaining controlled computational cost. This property is particularly relevant for medical imaging tasks involving complex anatomical structures and notable variations in lesion scale.

**Fig. (6):**
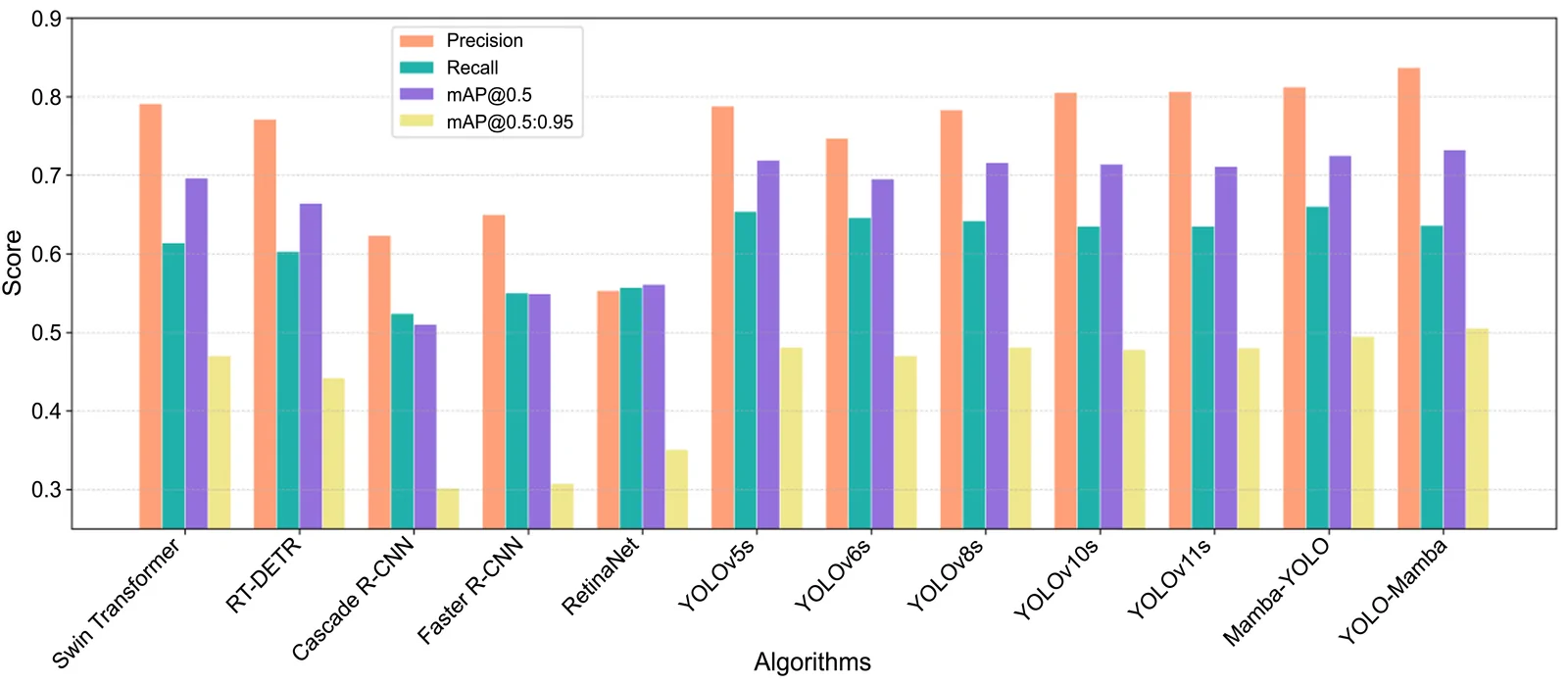
Performance comparison of different detection algorithms.

Figure **6** presents a comparison of different detection algorithms, focusing on four core performance metrics: Precision, Recall, mAP@0.5, and mAP@0.5:0.95. The bar chart clearly illustrates the performance distribution across all evaluated models, YOLO11-Mamba and Mamba-YOLO consistently outperforming other frameworks across most metrics. Specifically, YOLO11-Mamba stands out with the highest Precision, mAP@0.5, and mAP@0.5:0.95. These results confirm that the improved model achieves better accuracy and detection performance.

### 4.2 Statistical Performance Analysis

As shown in Table **2**, YOLO11-Mamba consistently outperforms the baseline YOLO11 across all core metrics. In particular, YOLO11-Mamba achieves mAP@0.5 and mAP@0.5:0.95 scores of 0.732±0.006 and 0.501±0.004, respectively, compared with 0.710±0.011 and 0.480±0.008 for YOLO11. Paired t-tests were further conducted based on the three independent runs to assess statistical significance. The results show that YOLO11-Mamba has an average improvement of 2.23% in mAP@0.5 (*p*=0.017) and 2.03% in mAP@0.5:0.95 (*p*=0.048), both *p<*0.05.

### 4.3 Ablation Study

To verify the performance gains introduced by the proposed framework, a systematic ablation study was conducted. Comparative experiments were performed among the improved model, the benchmark model, models incorporating individual modules, and models with modules added incrementally to analyze the contribution of each component to overall performance. The ablation results are summarized in Table **3**.

As summarized in Table **3**, detection performance improves with the integration of different modules. Introducing EVSSBlock into the baseline, the YOLO11 model increases mAP@0.5 and mAP@0.5:0.95 by 1.5% and 1.3%, respectively, indicating its contribution to feature representation. Further, incorporating DySample, it achieves mAP@0.5 and mAP@0.5:0.95 values of 0.715 and 0.486 without increasing model parameters. When EVSSBlock and DySample are combined, performance further improves to 0.728 and 0.500, with moderate increases in model size and computational cost.

With all three modules enabled, the model achieves its best overall performance, reaching mAP@0.5 and mAP@0.5:0.95 values of 0.732 and 0.505, respectively. These results suggest that the proposed modules provide complementary benefits and jointly enhance detection performance in renal tumor and cyst detection tasks.

### 4.4 Comparative Experiment on Attention Mechanisms

To further evaluate the effectiveness of the MLCA module designed in this paper for model performance enhancement, comparative experiments were conducted using several representative attention mechanisms. Specifically, SE, CBAM, and ECA were embedded into the YOLO11 network for comparison. All experiments were performed under identical network architectures and training settings to ensure a fair evaluation based on controlled variables. The comparison results are shown in Table **4**.

As demonstrated by the results in Table **4**, the MLCA attention module exhibits clear advantages across multiple performance metrics. Compared with the ECA attention module, MLCA achieves a 1.4% improvement in mAP@0.5 under the same parameter setting, indicating a substantial enhancement in detection accuracy. Moreover, although MLCA has fewer parameters than both the SE and CBAM attention mechanisms, it attains the highest mAP@0.5 value of 0.732. These findings indicate that the MLCA module has superior parameter efficiency while achieving optimal detection performance, providing a favorable balance between accuracy and computational efficiency.

### 4.5 Visual Analysis

Visualization is essential for understanding the training dynamics and performance characteristics of YOLO11-Mamba. As shown in Fig. (**7**), both the training and validation losses show a steady lower trend as the number of epochs grows, demonstrating that the model can effectively learn discriminative features while maintaining stable convergence. Although some variations are noted in the validation loss during the early training stages, the curve becomes smooth and stable in later epochs, indicating that the model is not severely overfitted and has strong generalization capacity.

As shown in Fig. (**8**), the detection performance assessed by mAP@0.5 and mAP@0.5:0.95 gradually increases with training and eventually converges. The rapid increase in mAP@0.5 suggests high object detection capabilities, but the continual improvement of mAP@0.5:0.95 reflects robust localization performance under stricter IoU criteria. Overall, these results show that YOLO11-Mamba has efficient learning behavior and strong convergence qualities during training.

**Fig. (9):**
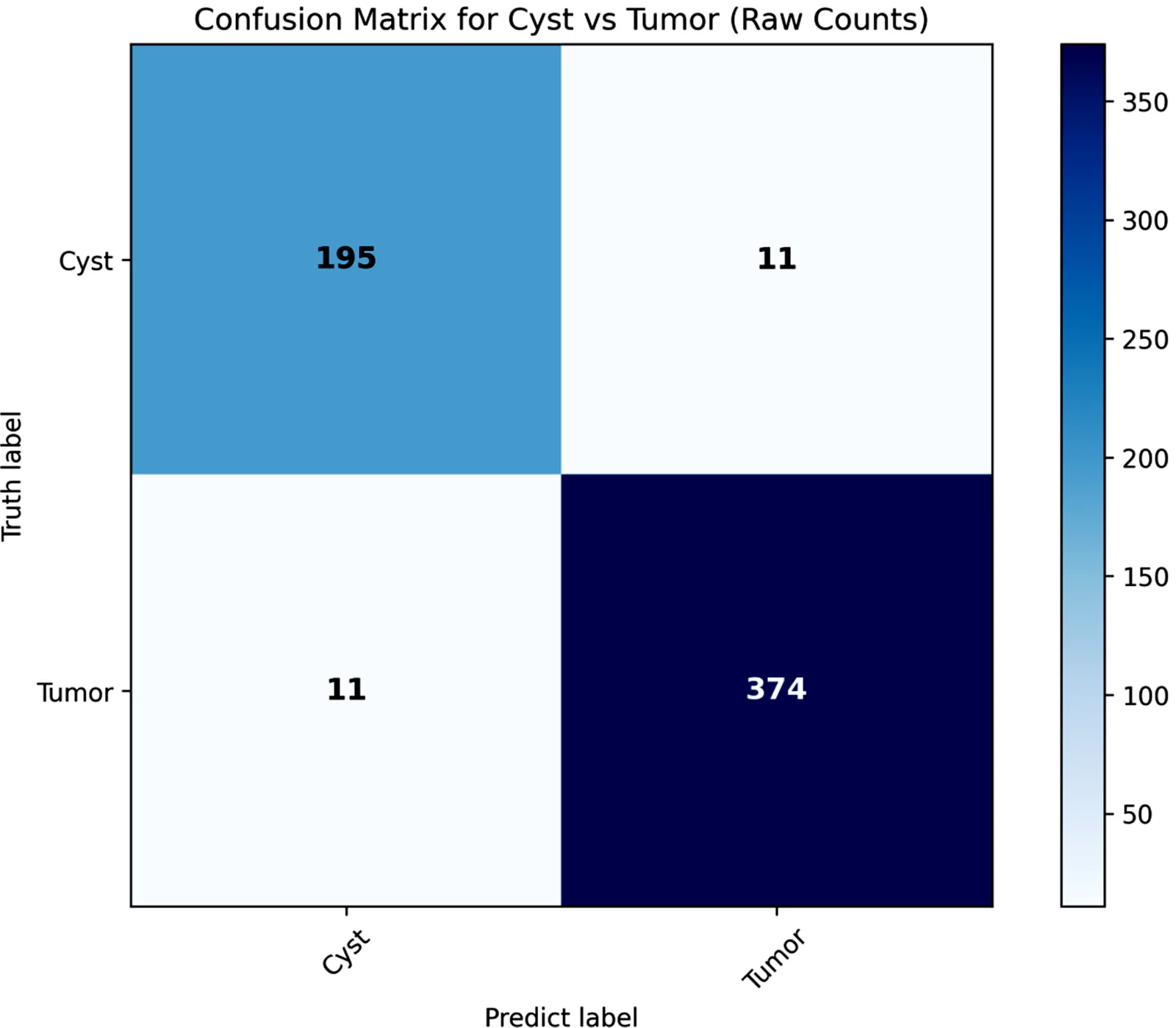
Confusion matrix.

Figure **9** depicts the confusion matrix utilized for model performance analysis. In cases of cysts, 195 samples were correctly classified (68%), while 11 samples (3%) were misclassified as tumors. For tumor cases, 374 samples were accurately classified (77%), while 11 samples (2%) were incorrectly categorized as cysts. The background category is not included in the confusion matrix because it is not clearly represented in the detection framework. As a result, this matrix only shows the performance and error distribution at the lesion level.

To visually compare the detection performance of YOLO11-Mamba and YOLO11, representative samples from the test set were selected for visualization. The results are shown in Fig. (**10**).

In Fig. (**10a**), the detection results of YOLO11 are shown, while Fig. (**10b**) shows the results produced by the proposed model. As shown in Fig. (**10**), when the image contains only a single lesion type (either a tumor or a cyst), the YOLO11-Mamba demonstrates improved detection performance compared with YOLO11. For cases with multiple lesion regions, the YOLO11-Mamba enables more accurate and stable detection of subtle tumor and cyst regions. These results indicate improved localization capability and a more reliable confidence score.

In object detection tasks, heatmap visualization provides an intuitive representation of the spatial attention distribution of different models by illustrating variations in detection confidence across image regions. Gradient-weighted Class Activation Mapping (Grad-CAM) is a gradient-based visualization technique that highlights the regions contributing most to a specific class prediction in deep neural networks [20]. To further analyze the effects of the proposed improvements, a heatmap visualization was employed to examine the output feature maps. Fig. (**11a-c**) presents a comparison of the predicted target locations and attention distributions generated by the YOLO11-Mamba model and the YOLO11. As shown in Fig. (**11c**), the proposed model exhibits more focused activation within lesion regions, characterized by concentrated high-response areas, while background regions show lower activation levels. Compared with the YOLO11, the proposed model demonstrates more precise attention in small lesion areas, which contributes to reduced missed detections and improved localization performance.

From a clinical standpoint, the YOLO11-Mamba model has better detection potential. Through result visualization and Grad-CAM heatmap analysis, this model provides assistance in the diagnosis of kidney tumors and cysts, thereby aiding radiologists in their diagnostic decisions, rather than replacing human judgment. These visual outputs enhance the transparency of the model and may reduce cognitive burden during diagnosis. In terms of practical deployment, the proposed framework is compatible with integration into existing PACS workflows. The model can process standard CT images and provide detection results as visual overlays (*e.g*., bounding boxes or heatmaps), making it suitable for use as a secondary reading aid in conventional radiology practice.

Moreover, regarding radiologist acceptance, the model offers intuitive and concise cues while maintaining competitive detection performance, which may help reduce diagnostic time and lower the risk of missed diagnoses. Based on the confusion matrix analysis, the false-positive burden remains within an acceptable level, indicating that the system is unlikely to excessively disrupt workflow.

### 4.6 Study Limitations

This study involved several limitations. First, the experiments were conducted exclusively on the publicly available KiTS23 dataset. Although KiTS23 is a well-established benchmark, future work will consider larger and more diverse multi-center datasets to further evaluate the generalizability of the proposed method. Second, the proposed approach is based on a two-dimensional slice-level detection strategy that does not fully exploit the full three-dimensional volumetric information inherent in CT data. As a result, spatial dependencies between adjacent slices and the three-dimensional structural characteristics of renal lesions are not explicitly modeled. Future research will explore the integration of three-dimensional detection frameworks or multi-slice feature fusion strategies to better leverage volumetric context and improve lesion representation. Although the incorporation of the Mamba mechanism improves detection accuracy, it also increases the number of model parameters and computational complexity, which affects inference efficiency. As shown in Table **1**, YOLO11-Mamba exhibits a lower inference speed compared with the YOLO11s. This indicates that the current design, based on state space, improves accuracy but introduces extra computational cost. This method may limit its use in real-time clinical applications with limited resources. Future work will focus on model lightweight, structural pruning, and hardware optimization to better balance accuracy and inference efficiency.

## CONCLUSION

To address the challenge of low detection accuracy due to blurred boundaries between renal tumors and cysts in anatomically complex abdominal CT images, this study proposes an improved YOLO11-Mamba model for renal lesion detection. An EVSSBlock module is designed to integrate the local feature extraction capability of conventional convolution with the long-range dependency modeling ability of Mamba, thereby enhancing the model’s representation of clinically relevant regions. In addition, a DySample upsampling module is introduced to improve feature fusion, and a mixed local channel attention mechanism is incorporated before the detection head to integrate multi-dimensional information and improve lesion localization. Comparative experiments conducted on a public dataset demonstrate the effectiveness of the proposed YOLO11-Mamba model in accurately detecting renal tumors and cysts in CT images.

## Figures and Tables

**Fig. (1):**
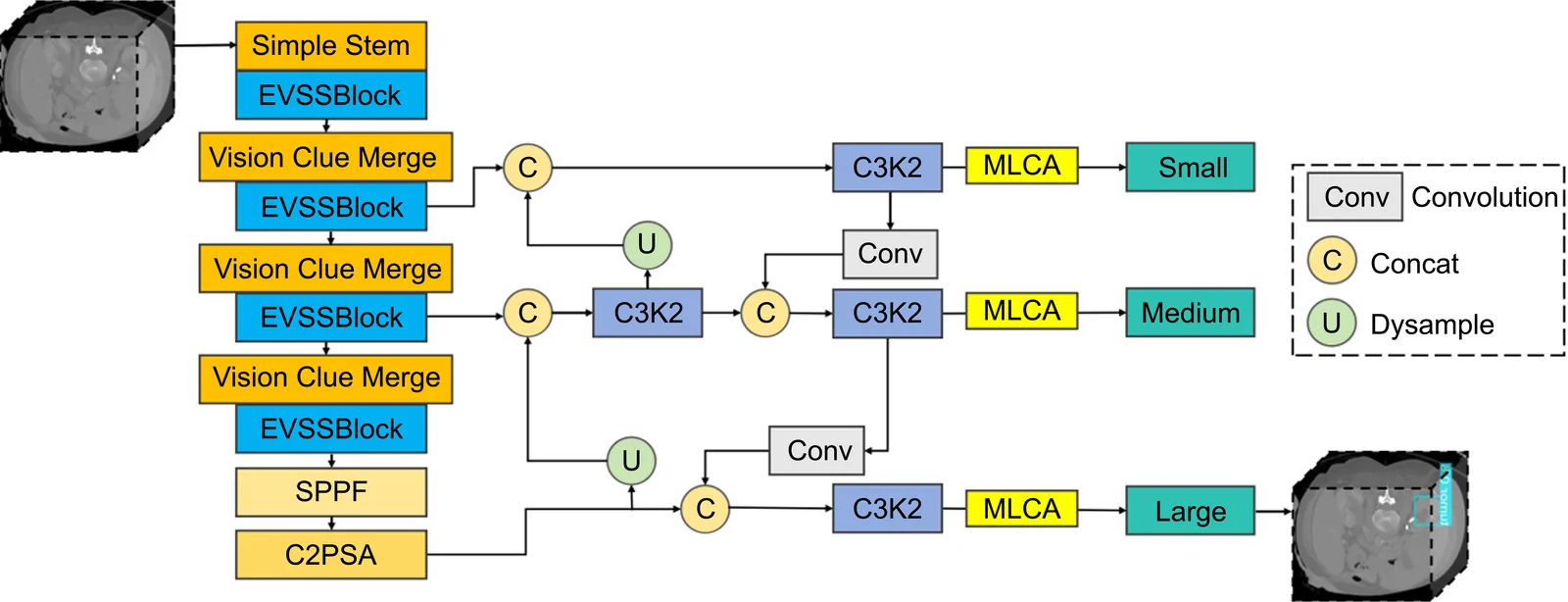
YOLO11-Mamba model.

**Fig. (2):**
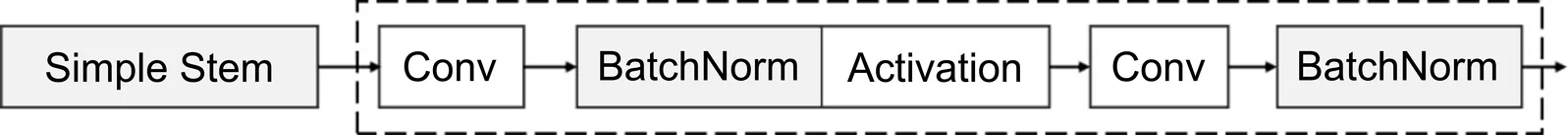
Stem model.

**Fig. (3):**
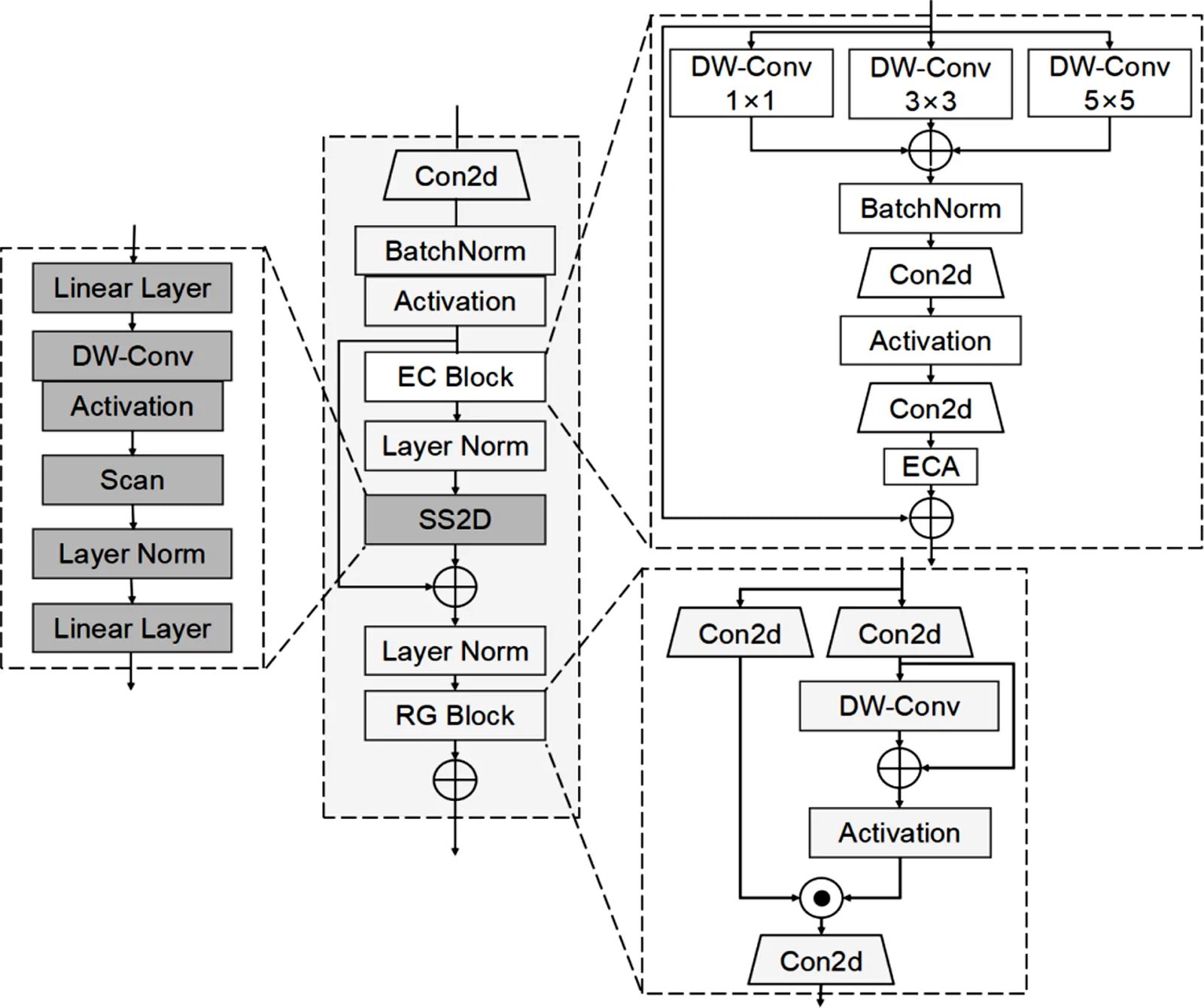
EVSSBlock module.

**Fig. (4):**
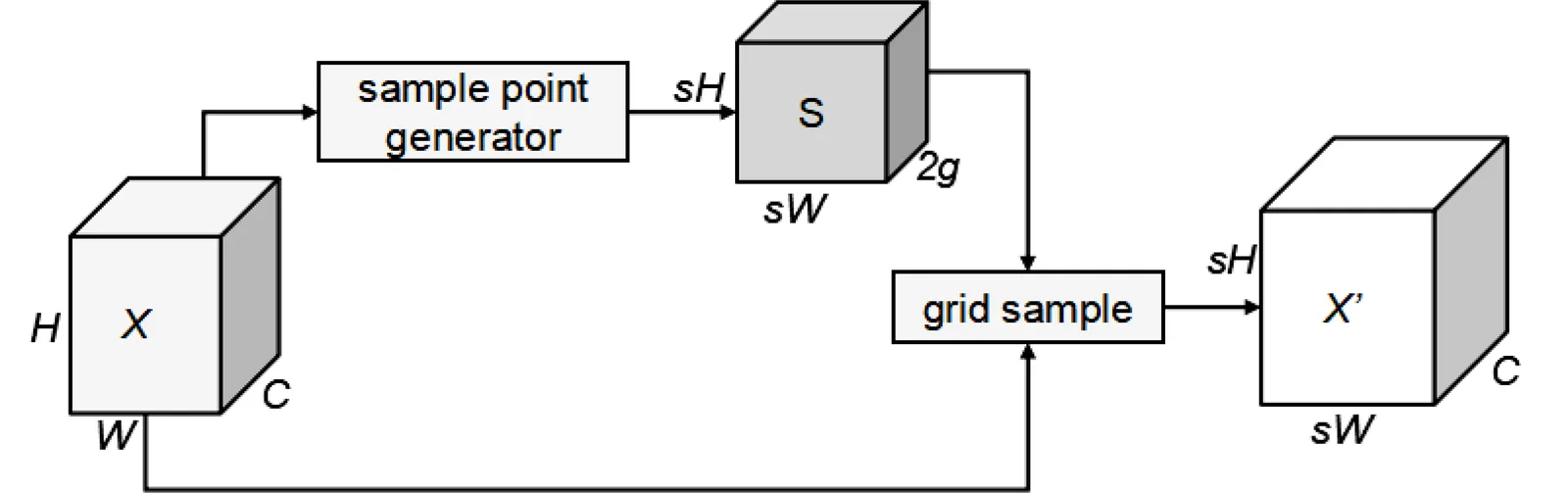
Dysample module.

**Fig. (5):**
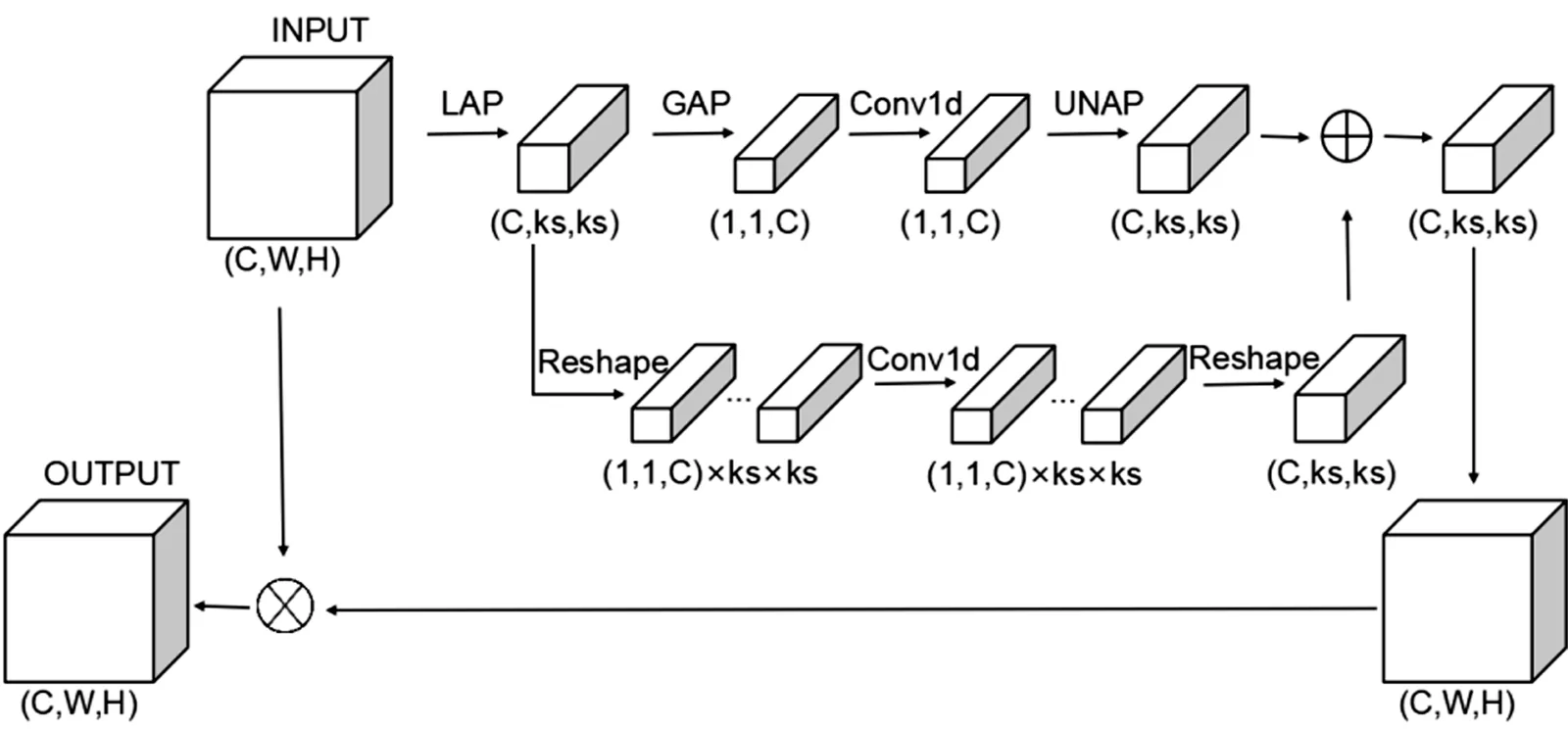
MLCA Module.

**Fig. (7):**
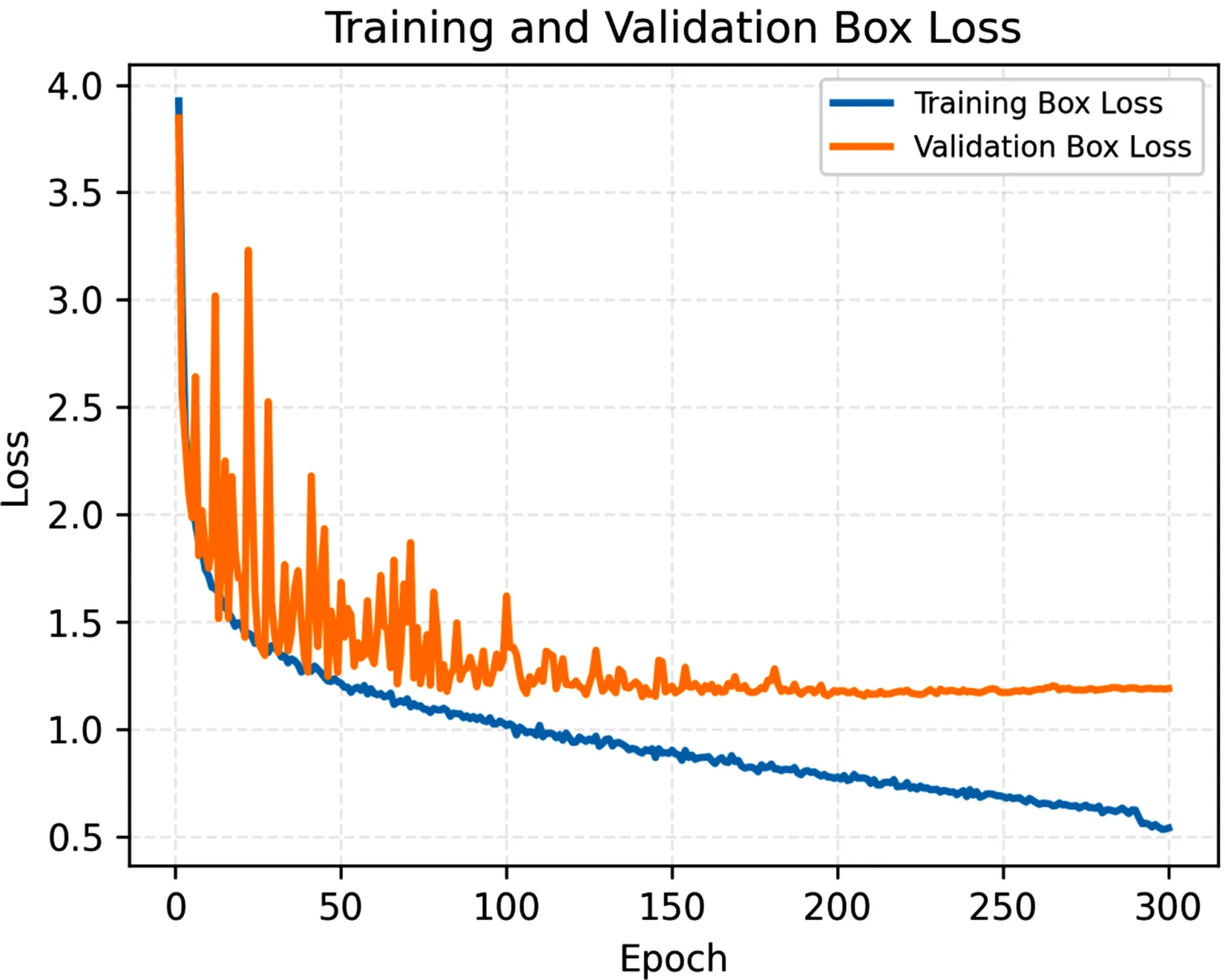
Graphical representation of training and validation loss.

**Fig. (8):**
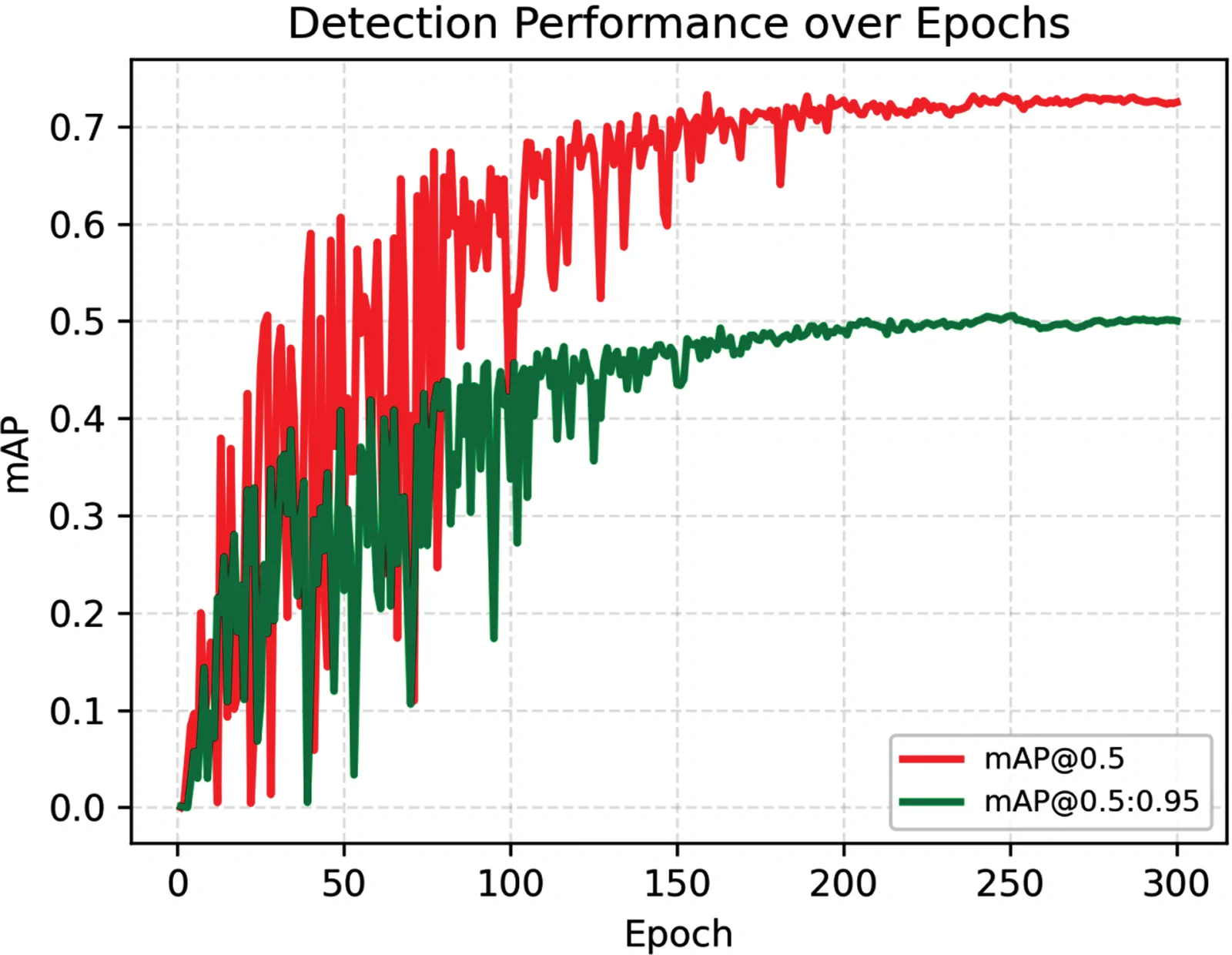
Graphical representation of a map.

**Fig. (10a, b):**
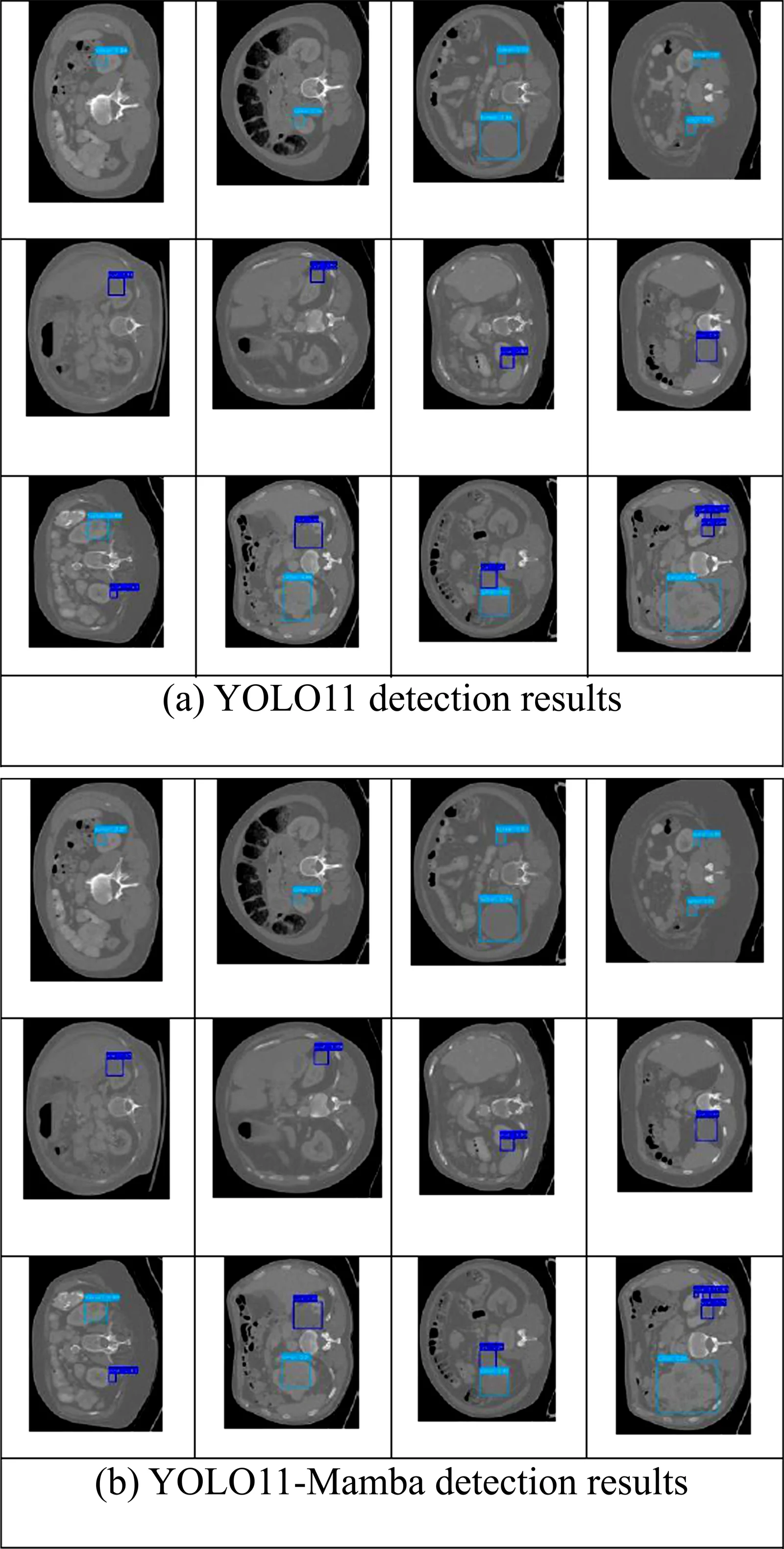
Comparison of baseline model and the improved model detection results.

**Fig. (11a-c):**
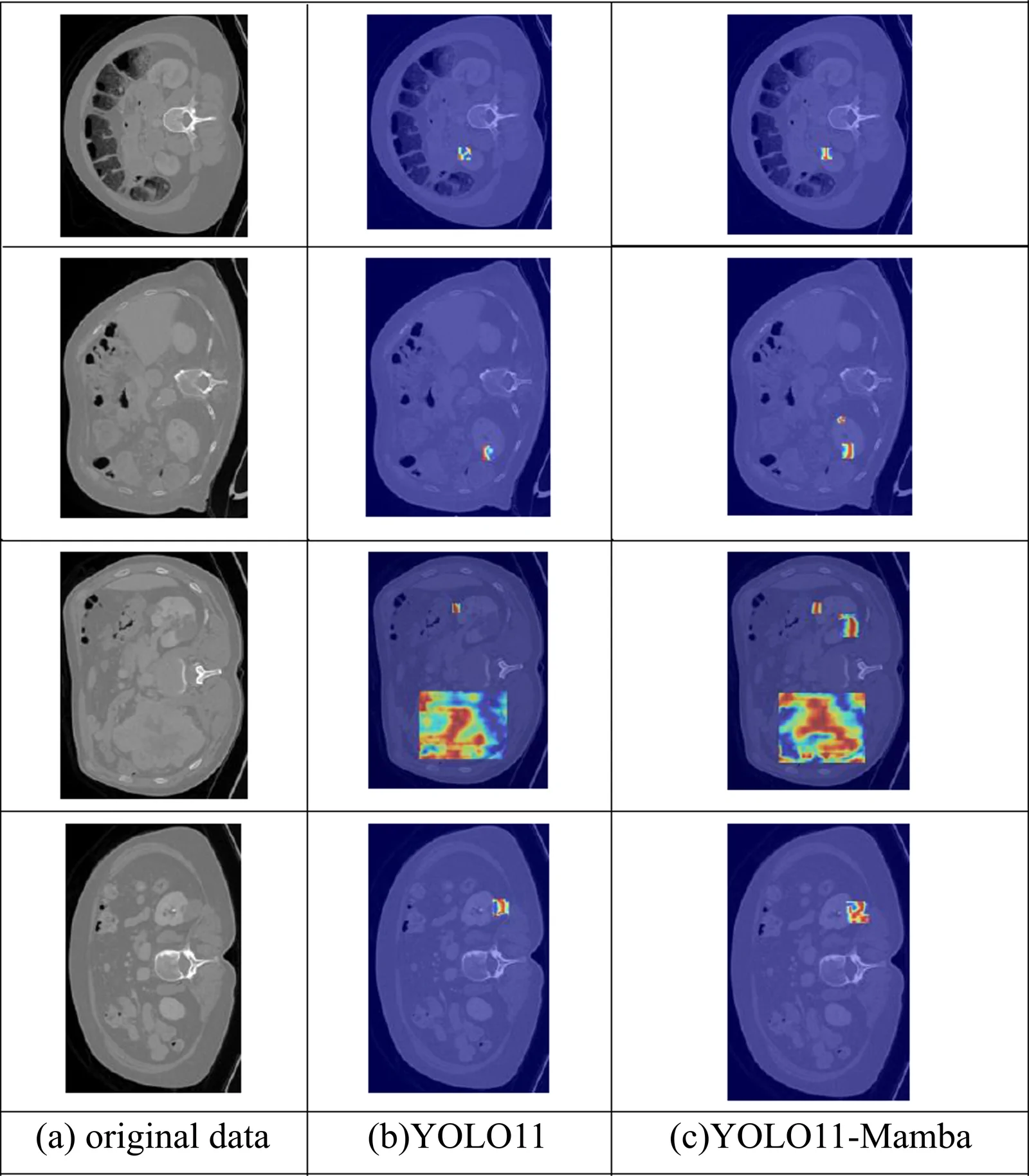
Comparison of heatmaps.

**Table 1:** Comparison of different models.

**Algorithm**	**Size**	**GFLOPs**	**FPS**	**Precision**	**Recall**	**mAP@0.5**	**mAP@0.5:0.95**
SwinTransformer-YOLO	67.80M	407.4G	106.38	0.791	0.614	0.696	0.470
RT-DETR	66.10M	108.0G	99.01	0.771	0.603	0.664	0.442
Cascade-RCNN	69.16M	162.0G	**45.60**	0.623	0.524	0.510	0.302
Faster-RCNN	41.35M	134.0G	53.00	0.650	0.550	0.549	0.308
RetinaNet	36.35M	128.0G	55.10	0.553	0.557	0.561	0.351
YOLOv5s	18.60M	24.0G	370.37	0.788	0.654	0.719	0.481
YOLOv6s	32.90M	44.0G	357.14	0.747	0.646	0.695	0.470
YOLOv8s	21.50M	28.8G	256.41	0.783	0.642	0.716	0.481
YOLOv10s	**16.50M**	24.8G	357.14	0.805	0.635	0.714	0.478
YOLO11s	19.20M	**21.3**G	312.50	0.806	0.635	0.711	0.480
Mamba-YOLO [13]	21.60M	49.7G	159.75	0.812	**0.660**	0.725	0.495
YOLO11-Mamba	30.00M	32.9G	105.90	**0.837**	0.636	**0.732**	**0.505**

**Table 2:** Statistical performance comparison across three independent runs.

**Algorithm**	**Size**	**GFLOPs**	**FPS**	**Precision**	**Recall**	**mAP@0.5**	**mAP@0.5:0.95**
YOLO11s	19.20M	21.3G	312.50	0.806 ± 0.003	0.632 ± 0.013	0.710 ± 0.011	0.480 ± 0.008
YOLO11-Mamba	30.00M	32.9G	105.90	0.837 ± 0.002	0.644 ± 0.007	0.732 ± 0.006	0.501 ± 0.004

**Table 3:** Ablation experimental results.

**YOLO11s**	**ESSBlock**	**Dysample**	**MLCA**	**Size**	**FLOPs**	**FPS**	**mAP@0.5**	**mAP@0.5:0.95**
√	-	-	-	**19.20M**	**21.3G**	312.50	0.711	0.480
√	√	-	-	30.00M	32.7G	122.82	0.726	0.493
√	-	√	-	19.20M	21.6G	332.58	0.715	0.486
√	-	-	√	19.20M	21.7G	256.41	0.721	0.483
√	√	√	-	30.00M	32.8G	113.27	0.728	0.500
√	√	√	√	30.00M	32.9G	**105.90**	**0.732**	**0.505**

**Table 4:** Comparison of model performance using different attention.

**Attention Models**	**Size**	**FLOPs**	**FPS**	**mAP@0.5**
MLCA	**30.00M**	32.9G	105.90	**0.732**
SE	30.10M	**32.8G**	109.48	0.708
CBAM	30.10M	32.9G	**103.87**	0.723
ECA	30.00M	32.8G	104.00	0.718

## Data Availability

This study uses data from [KiTS23], available at [neheller/kits23: The official repository of the 2023 Kidney Tumor Segmentation Challenge (KiTS23)]. Researchers can access the dataset freely under the conditions specified by the data provider.

## LIST OF ABBREVIATIONS

CT: Computed Tomography
YOLO11: You Only Look Once Version 11
DySample: Dynamic Upsampling Module
MLCA: Multi-Dimensional Local Channel Attention
SE: Squeeze-and-Excitation
ECA: Efficient Channel Attention

## References

[1] Aljohani M. (2025). Clinical-oriented hierarchical machine learning framework for early kidney tumor detection and malignant subtype classification.. Tomography.

[2] Zhang Z., Sun G., Zheng K., Yang J.K., Zhu X., Li Y. (2023). TC-Net: A joint learning framework based on CNN and vision transformer for multi-lesion medical images segmentation.. Comput. Biol. Med..

[3] Zhao Z., Chen H., Li J., Wang L. (2022). Boundary attention U-net for kidney and kidney tumor segmentation.. Annu. Int. Conf. IEEE Eng. Med. Biol. Soc..

[4] Praveen S.P., Sidharth S.R., Priya T.K., Kavuri Y.S., Sindhura S.M., Donepudi S. Resnet and resnext-powered kidney tumor detection: A robust approach on a subset of the kauh dataset.. Conference: 2023 2nd International Conference on Automation, Computing and Renewable Systems (ICACRS).

[5] Alzu’bi D., Abdullah M., Hmeidi I., AlAzab R., Gharaibeh M., El-Heis M., Almotairi K.H., Forestiero A., Hussein A.M., Abualigah L. (2022). Kidney tumor detection and classification based on deep learning approaches: A new dataset in CT scans.. J. Healthc. Eng..

[6] Bingol H., Yildirim M., Yildirim K., Alatas B. (2023). Automatic classification of kidney CT images with relief based novel hybrid deep model.. PeerJ Comput. Sci..

[7] Zabihollahy F., Schieda N., Krishna S., Ukwatta E. (2020). Automated classification of solid renal masses on contrast-enhanced computed tomography images using convolutional neural network with decision fusion.. Eur. Radiol..

[8] Fouziya M., Reddy J.S., Reddy A.A., Mohammad Bilal J., Kumar K.S., Rout S.K. Unveiling kidney tumor detection using cutting edge deep learning object detection techniques.. 2024 IEEE 6th International Conference on Cybernetics, Cognition and Machine Learning Applications (ICCCMLA),.

[9] Anari P.Y., Lay N., Zahergivar A., Firouzabadi F.D., Chaurasia A., Golagha M., Singh S., Homayounieh F., Obiezu F., Harmon S., Turkbey E., Merino M., Jones E.C., Ball M.W., Linehan W.M., Turkbey B., Malayeri A.A. (2024). Deep learning algorithm (YOLOv7) for automated renal mass detection on contrast-enhanced MRI: A 2D and 2.5D evaluation of results.. Abdom. Radiol..

[10] Pande S.D., Agarwal R. (2024). Multi-class kidney abnormalities detecting novel system through computed tomography.. IEEE Access.

[11] Gu A., Dao T. (2023). Mamba: Linear-time sequence modeling with selective state spaces.. arXiv.

[12] Jocher G., Qiu J. (2024). Ultralytics YOLO11[EB/OL]. (2024-09- 30) [2025-06-19].. https://github.com/ultralytics/ultralytics.

[13] Liu Y., Tian Y., Zhao Y., Yu H., Xie L., Wang Y., Ye Q., Jiao J., Liu Y. Vmamba: Visual state space model.. Advances in Neural Information Processing Systems 37,.

[14] Chen CS, Chen GY, Zhou D (2024). Res-vmamba: Fine-grained food category visual classification using selective state space models with deep residual learning.. arXiv.

[15] Wang Z., Li C., Xu H., Zhu X., Li H. (2025). Mamba yolo: A simple baseline for object detection with state space model.. Proc. Conf. AAAI Artif. Intell..

[16] Cao Y., Zhang J., Zhuo R., Zhao J., Dong Y., Liu T., Zhao H. (2024). BrYOLO-mamba: A approach to efficient tracheal lesion detection in bronchoscopy.. IEEE Access.

[17] Liu W., Lu H., Fu H., Cao Z. Learning to upsample by learning to sample.. 2023 IEEE/CVF International Conference on Computer Vision (ICCV).

[18] Wan D., Lu R., Shen S., Xu T., Lang X., Ren Z. (2023). Mixed local channel attention for object detection.. Eng. Appl. Artif. Intell..

[19] Heller N, Isensee F (2023). The state of the art in kidney and kidney tumor segmentation: Results of the KiTS23 Challenge.. Med. Image Anal..

[20] Zhang H., Zhao S., Song Y., Ge S., Liu D., Yang X., Wu K. (2022). A deep learning and Grad-Cam-based approach for accurate identification of the fall armyworm (*Spodoptera frugiperda*) in maize fields.. Comput. Electron. Agric..

